# Risk of Hospitalized Falls and Hip Fractures in 22,103 Older Adults Receiving Mental Health Care vs 161,603 Controls: A Large Cohort Study

**DOI:** 10.1016/j.jamda.2020.03.005

**Published:** 2020-12

**Authors:** Brendon Stubbs, Gayan Perara, Ai Koyanagi, Nicola Veronese, Davy Vancampfort, Joseph Firth, Katie Sheehan, Marc De Hert, Robert Stewart, Christoph Mueller

**Affiliations:** a South London and Maudsley NHS Foundation Trust, Denmark Hill, London, United Kingdom; b Institute of Psychiatry, Psychology and Neuroscience (IoPPN), King's College London, London, United Kingdom; c Research and Development Unit, Parc Sanitari Sant Joan de Déu, Universitat de Barcelona, Fundació Sant Joan de Déu, CIBERSAM, Barcelona, Spain; d ICREA, Barcelona, Spain; e Primary Care Department, Azienda ULSS (Unità Locale Socio Sanitaria) 3 “Serenissima,” Dolo, Venice, Italy; f Department of Rehabilitation Sciences, KU Leuven–University of Leuven, Leuven, Belgium; g University Psychiatric Centre, KU Leuven, University of Leuven, Kortenberg, Belgium; h NICM Health Research Institute, Western Sydney University, Sydney, New South Wales, Australia; i Division of Psychology and Mental Health, Faculty of Biology, Medicine and Health, University of Manchester, Manchester, United Kingdom; j Department of Population Health Sciences, School of Population Health & Environmental Sciences, King's College London, London, United Kingdom; k University Psychiatric Centre KU Leuven, Kortenberg, Belgium; l Antwerp Health Law and Ethics Chair, University of Antwerp, Antwerp, Belgium

**Keywords:** Hip fracture, falls dementia, mental illness, schizophrenia, substance use disorder

## Abstract

**Objectives:**

To investigate the risk of hospitalized fall or hip fracture among older adults using mental health services.

**Design:**

Retrospective cohort study.

**Setting and Participants:**

Residents of a South London catchment aged >60 years receiving specialist mental health care between 2008 and 2016.

**Measures:**

Falls and/or a hip fracture leading to hospitalization were ascertained from linked national records. Incidence rates and incidence rate ratios (IRRs) were age- and gender-standardized to the catchment population. Multivariable survival analyses were applied investigating falls and/or hip fractures as outcomes.

**Results:**

In 22,103 older adults, incidence rates were 60.1 per 1000 person-years for hospitalized falls and 13.7 per 1000 person-years for hip fractures, representing standardized IRRs of 2.17 [95% confidence interval (CI) 2.07-2.28] and 4.18 (3.79-4.60), respectively. The IRR for falls was high in those with substance-use disorder [IRR = 6.72 (5.35-8.33)], bipolar disorder [IRR = 3.62 (2.50-5.05)], depression [IRR = 2.28 (2.00-2.59)], and stress-related disorders [IRR = 2.57 (2.10-3.11)]. Hip fractures were increased in all populations (IRR > 2.5), with greatest risk in substance use disorders [IRR = 12.64 (7.22-20.52)], dementia [IRR = 4.38 (3.82-5.00)], and delirium [IRR = 4.03 (3.00-5.29)]. Comparing mental disorder subgroups with each other, after the adjustment for 25 potential confounders, patients with dementia and substance use had a significantly increased risk of falls, and patients with dementia also had an increased risk of hip fractures.

**Conclusion and Implications:**

Older people using mental health services have more than double the incidence of falls and 4 times the incidence of hip fractures compared to the general population. Although incidences differ between diagnostic subgroups, all groups have a higher incidence than the general population. Targeted interventions to prevent falls and hip fractures among older adult mental health service users are urgently needed.

Falls are common in older adults^1^ and associated with substantial morbidity, reduced quality of life, substantial health care expenditure, and premature mortality.^2^, ^3^, ^4^ Falls are a leading cause of hip fractures, which are associated with pronounced disability^5^ and reduced functional capacity.^6^ Research in the general population has suggested that between 27% and 59% of older people transition into permanent long-term care within the first year after a hip fracture.^7^^,^^8^ Moreover, hip fractures are associated with increased mortality.^9^, ^10^, ^11^ Unsurprisingly, understanding and preventing falls and hip fractures is a global health priority.^12^

Older adults using mental health services are at a particularly increased risk of experiencing falls and hip fractures due to a range of risk factors.^13^^,^^14^ For instance, people with late-life cognitive and functional mental disorders have increased risk of physical multi- and comorbidities,^15^, ^16^, ^17^, ^18^, ^19^, ^20^ polypharmacy,^21^ use of individual medications (eg, antipsychotic medication,^22^ antidepressants^23^), and impaired cognition. Moreover, these populations often have other risk factors such as inadequate nutrition,^24^ physical inactivity,^25^ impaired physical performance,^26^^,^^27^ and high smoking rates.^28^ Previous research has demonstrated that mental health service users including people with dementia, psychotic disorders, bipolar disorder, anxiety- and stress-related disorders, and major depression have increased risk of osteoporosis compared with age- and sex-matched controls and are thus at greater risk of hip fractures.^29^^,^^30^ Falls are the leading cause of patient safety incidents reported in older adult mental health services.^31^

Despite the aforementioned, minimal representative research has considered and compared which mental disorder groups are at greatest risk. A systematic review identified that people with dementia are at increased risk of falls, but all the sample sizes included fewer than 300 people.^32^ Two cohort studies suggested that people with dementia are at increased risk of fractures.^33^^,^^34^ Falls risk in late-life psychotic disorders has received very little investigation, although recent cohort studies have suggested that this population is at increased risk of fractures.^35^^,^^36^ A recent systematic review suggested that people with depressive symptoms are at increased risk of hip fractures, although relatively few (n = 5) studies included people with confirmed depression.^37^ A meta-analysis suggested that people with major depression were at increased risk of future falls, but the sample size was small (n = 965).^38^ Minimal information is available on falls and hip fractures in older adults with bipolar disorder, substance use disorder, or anxiety- and stress-related disorders to date.^39^

In light of the above, we assembled a cohort study from routine health care data for a catchment population of 1.3 million with the following aims: (1) to compare the incidence of falls and/or hip fractures among older patients with diagnosed mental disorders to that in the general population; and (2) to compare risk of falls and/or hip fracture for diagnostic subgroups to establish if any particular group of mental health conditions was at greatest risk of a hospitalized fall or hip fracture.

## Methods

### Study Setting and Data Source

A retrospective observational study was conducted using data from the South London and Maudsley NHS Foundation Trust (SLaM) Biomedical Research Centre (BRC) Case Register. SLaM serves a geographic catchment of 4 South London boroughs with a population in excess of 1.3 million residents. The data for the current study were assembled from the Clinical Record Interactive Search (CRIS) resource, which renders a deidentified version of SLaM's routine electronic health record accessible for research projects within a robust and patient-led governance framework.^40^ The SLaM BRC Case Register has been described in detail.^34^^,^^36^^,^^41^, ^42^, ^43^, ^44^, ^45^, ^46^ Data are currently archived in CRIS on more than 400,000 people with a range of mental disorders and the database has full approval for secondary analysis (Oxford Research Ethics Committee C, reference 18/SC/0372).

### Participants

All SLaM patients aged 60 years or older who received a specific diagnosis of a mental or behavioral disorder according to International Classification of Diseases–10th Revision (ICD-10)^47^ chapter 5 between January 1, 2008 and December 31, 2016 were included. Dates of diagnosis and ICD-10 code were obtained from a structured field, and the first diagnosis date after the age of 60 years served as the index date for defining both the inclusion criterion and covariates. We categorized these index diagnoses according to the following ICD-10 groups: dementia (F00-F03), delirium (F05), mild cognitive impairment (MCI; F06.7), substance use disorder (F10-F19), psychotic disorder (F20-29), bipolar affective disorder (F30, F31), unipolar depression (F32, F33), any anxiety disorder (F40, F41), or a stress-related disorder (F43.0, F43.2, F43.8, F43.9, an exceptionally stressful life event producing an acute stress reaction or a significant life change leading to continued unpleasant circumstances that result in an adjustment disorder). All remaining diagnoses were grouped in the “other category.” SLaM patient records have been linked with Hospital Episode Statistics (HES), a database of all hospital care received in England,^48^ which were available until March 31, 2016 at the time of analysis. From available HES data, we further generated a population control data set for all residents in the catchment area, ascertaining all admissions for falls (W00-W19) and/or hip fractures (S72) for the catchment in the year 2011, and applying 2011 national census data for the over-60s population in this catchment to derive denominators.^49^^,^^50^

### Outcomes

The co-primary outcomes were (1) a fall leading to hospitalization and (2) a hip fracture. Admission due to a fall was classified if an ICD-10 code of W00-19 was recorded as any discharge diagnosis. For hip fractures, we identified hospitalizations that contained the ICD-10 code S72 among the discharge diagnoses. We ascertained all falls and hip fractures occurring within the first 12 months after the index date, and then additionally followed all patients to a censoring point defined as the first of the following: fall or hip fracture, death, or March 31, 2016.

### Covariates

Covariates included age, gender, neighborhood deprivation level (based on index of multiple deprivation applied to the lower super output area geographic unit containing the patient's address^51^), ethnicity (white vs nonwhite), and cohabiting status, dichotomized into cohabiting (civil partnership, married, cohabiting) and noncohabiting (single, divorced, civil partnership dissolved, widowed, separated) groups. Mental and physical health, as well as functioning, was determined from the Health of the Nation Outcome Scales.^52^ We extracted the following Health of the Nation Outcome Scales subscale scores: (1) overactive or aggressive behavior, (2) nonaccidental self-injury, (3) problem-drinking or drug taking, (4) hallucinations or delusions, (5) depressed mood, (6) physical illness or disability, (7) relationship problems, (8) activities of daily living (ADL) problems, (9) living conditions, and (10) occupational and recreational activity limitations. Scores for each item range from 0 (no problem) to 4 (severe problem). For physical illness we created an ordinal scale (0-1 = no or minor problem; 2 = mild problem; 3-4 = moderate to severe problem) and dichotomized the scores of all other scales into “no or mild problem” (score 0-1) and “problem present” (score 2-4).

Lastly, we ascertained the medications from structured medication fields supplemented by natural language processing applications applied to free text.^40^ The presence of the following medication groups was extracted for the period of 6 months before and after the index date, including medications for osteoporosis, calcium/vitamin D supplementation, anticholinergics, analgesics, hypnotics (including anxiolytics and benzodiazepines), antihypertensives, antipsychotics, and antidepressants.

### Statistical Analysis

We used Stata, version 13, software. First, we ascertained the number of falls or hip fractures in the first year after the initial diagnostic statement in the whole patient cohort and diagnostic subgroups. Age- and gender-standardized incidence rates of these outcomes were calculated in relation to the English Standard Population both for the patient and general population cohorts. We calculated indirectly standardized incidence rate ratios (IRRs) comparing number of outcomes in the patient to the expected number of outcomes in the general population according to 5-year age-gender bands. Second, we compared the 10 diagnostic categories in terms of demographic, symptom, functioning, and medication prescription medication baseline variables. Thereafter, we constructed Cox proportional hazard models comparing each condition to the remainder of the sample with first hospitalized fall or first hospitalized hip fracture as the outcome variable. In total, 31% of patients in our sample had missing data on at least 1 of the covariates. As we judged missingness in this sample to be random, we imputed missing values using chained equations to maximize statistical power.^53^ Using the *mi* package in Stata, we created 5 imputed data sets by replacing missing values through simulated values assembled from potential covariates and outcome values. Rubin's rules^54^ were applied to combine coefficients in final analyses.

## Results

We extracted data on 22,103 patients aged 60 years with a specific mental disorder diagnosis in the observation period. The mean (SD) age of the sample was 77.0 (9.6) years and 57.3% were female. According to the index diagnosis, 34.6% had dementia (n = 7650), 9.8% delirium (n = 2158), 4.6% MCI (n = 1026), 5.2% substance use disorder (n = 1139), 8.0% psychotic disorder (n = 1777), 2.5% bipolar affective disorder (n = 542), 15.7% unipolar depression (n = 3462), 4.3% anxiety disorder (n = 942), 5.7% stress-related disorder (n = 1257), and 9.7% had another specified mental disorder (n = 2150).

Baseline characteristics for the sample are described in Table 1 according to diagnostic groups, including details of medication.

**Table 1:** Sample Characteristics for the Whole Cohort and by Diagnostic Group

Diagnoses	Whole Cohort (n = 22,103)	Dementia (n = 7650)	Delirium (n = 2158)	MCI (n = 1026)	Substance Use Disorder (n = 1139)	Psychotic Disorder (n = 1777)	Bipolar Affective Disorder (n = 542)	Unipolar Depression (3462)	Anxiety Disorder (n = 942)	Stress-Related Disorder (n = 1257)	Other (n = 2150)	*P* Value∗
Sociodemographic status and cognitive function†												
Mean age at diagnosis (SD)	77.0 (9.6)	82.1 (7.6)	80.9 (8.3)	79.3 (8.3)	66.6 (6.0)	71.6 (8.1)	70.2 (7.8)	75.2 (9.4)	74.4 (9.1)	76.3 (9.3)	69.8 (9.3)	<.001
Female gender	57.3	61.5	54.6	59.6	31.7	58.5	56.1	58.8	67.5	55.5	51.1	<.001
Nonwhite ethnicity	22.1	25.7	16.8	25.5	12.3	40.8	17.2	18.5	11.9	14.8	17.9	<.001
Married or cohabiting status	32.6	33.3	31.3	34.4	28.7	16.7	35.7	34.1	40.9	32.3	40.1	<.001
Mean index of deprivation (SD)	26.8 (11.7)	27.0 (11.5)	26.7 (11.8)	27.6 (11.1)	27.1 (11.5)	29.6 (11.1)	25.5 (12.2)	26.3 (11.9)	25.8 (11.9)	27.4 (11.8)	24.9 (12.3)	<.001
HoNOS symptoms or disorders†												
Overactive, aggressive behavior	21.6	23.9	40.0	6.0	25.3	23.7	28.5	12.2	13.9	10.4	21.0	<.001
Nonaccidental self-injury	4.6	1.4	2.2	0.5	11.0	3.6	2.6	13.0	5.4	10.8	5.9	<.001
Problem drinking or drug taking	5.1	3.2	3.6	3.6	73.3	4.6	5.7	5.0	3.8	5.0	4.8	<.001
Cognitive problems	56.2	88.7	76.1	45.4	41.9	24.0	17.8	20.6	11.9	20.4	34.3	<.001
Hallucinations or delusions	18.1	13.6	33.4	4.9	13.8	63.1	20.1	8.6	2.9	2.9	20.3	<.001
Depressed mood		13.1	17.7	13.5	28.3	14.0	25.2	75.4	45.3	52.2	32.4	<.001
HoNOS+ Physical illness or disability score—Comorbidity†												<.001
No or minor problem	35.3	41.6	9.6	46.1	40.9	51.3	47.8	30.0	38.3	15.3	36.2	
Mild problem	26.4	27.5	23.5	30.6	27.5	25.4	28.5	23.9	29.4	25.4	27.2	
Moderate-severe problem	38.3	30.9	66.9	23.3	31.6	23.3	23.7	46.1	32.3	59.3	36.6	
HoNOS functional problems†												
Relationship problem	22.1	18.3	26.2	10.3	34.7	35.5	29.5	21.8	21.1	17.3	33.5	<.001
Activities of daily living problem	58.0	66.9	76.6	31.4	51.9	41.9	35.0	51.3	39.8	61.9	44.0	<.001
Problem with living conditions	13.8	13.6	14.8	9.2	24.4	20.1	11.3	12.9	10.1	10.1	14.7	<.001
Problem with occupational and recreational activities	32.1	32.9	36.8	16.8	37.9	29.6	24.1	37.3	30.2	25.6	29.4	<.001
Medication prescription‡												
Osteoporosis medication	3.4	4.7	3.3	4.1	1.1	1.7	2.4	3.3	3.4	2.4	1.8	<.001
Calcium/vitamin D	6.5	5.9	12.7	3.7	3.6	6.2	5.7	6.6	6.5	8.9	3.7	<.001
Any anticholinergic	48.9	41.2	55.9	32.2	32.1	71.4	73.1	66.1	58.4	41.6	33.6	<.001
Analgesics	20.3	24.0	22.3	21.6	16.5	17.0	20.3	20.3	15.5	15.9	13.8	<.001
Hypnotics	18.5	13.0	21.0	6.3	20.2	25.0	41.9	25.2	33.1	14.2	13.9	<.001
Antihypertensives	27.0	32.2	26.7	34.8	14.6	26.2	23.8	28.5	25.9	18.1	16.1	<.001
Antipsychotics	21.1	14.4	29.5	3.3	7.9	74.2	59.4	17.9	12.6	5.0	16.7	<.001
Antidepressants	35.3	24.4	29.5	20.2	19.6	21.4	38.8	76.5	61.4	37.9	27.0	<.001
Hospitalized fall 1 year before index date	8.5	9.9	15.1	6.8	8.7	3.6	4.2	8.0	5.1	9.0	4.2	<.001

HoNOS, Health of the Nation Outcome Scales; SD, standard deviation.

Unless otherwise noted, values are percentages.

∗ *P* value for heterogeneity across diagnostic groups, calculated from chi^2^ tests or 1-way analysis of variance.

† At or closest to index date.

‡ Ascertained in a window of 1 year around the index date.

### Incidence of Hospitalized Falls in the Year After Index Date

In total, 1708 falls occurred in the patient cohort in the year after index date and the age-and-gender-standardized incidence rate was 60.1 [95% confidence interval (CI): 57.3-64.0] falls per 1000 person-years. In the general population cohort of 161,603 people over the age of 60 years in the year 2011, a total number of 3484 falls occurred. The age- and gender-standardized incidence rate was 22.1 (95% CI: 21.4-22.9) falls per 1000 person-years. Comparing the patient and the general population the age- and gender-adjusted IRR for falls was 2.17 (95% CI: 2.07-2.28). Incidence rates and ratios for falls and/or hip fractures according to diagnostic groups are presented in Table 2. People with delirium, substance use, and stress-related disorders had the highest age- and gender-adjusted incidence rates, and people with a psychotic or anxiety disorder the lowest. The highest increase of falls compared with the general population was detected in people with a substance use disorder (IRR: 6.72) and bipolar affective disorder (IRR: 3.62), and the lowest in MCI (IRR: 1.51) and anxiety disorder (IRR: 1.67).

**Table 2:** Incidence Rates and Ratios by Diagnostic Groups for Hospitalized Falls and/or Hip Fractures

	Falls		Hip Fractures	
	Age- and Gender-Standardized Incidence Rate∗	IRR Compared to the General Population†	Age- and Gender-Standardized Incidence Rate∗	IRR Compared to the General Population†
Dementia (n = 7650)	65.1 (54.0-76.8)	1.99 (1.85-2.14)	16.4 (12.2-21.0)	4.38 (3.82-5.00)
Delirium (n = 2158)	80.1 (59.4-103.0)	2.11 (1.84-2.42)	15.5 (7.0-26.1)	4.03 (3.00-5.29)
MCI (n = 1026)	45.7 (30.9-63.7)	1.51 (1.16-1.94)	10.9 (4.4-20.8)	2.45 (1.30-4.18)
Substance use disorder (n = 1139)	76.7 (54.9-102.3)	6.72 (5.35-8.33)	19.0 (8.7-33.9)	12.64 (7.22-20.52)
Psychotic disorder (n = 1777)	36.8 (27.6-48.0)	1.82 (1.40-2.32)	7.9 (4.3-13.4)	3.05 (1.67-5.12)
Bipolar affective disorder (n = 542)	61.0 (41.8-86.0)	3.62 (2.50-5.05)	—‡	7.58 (3.47-14.37)
Unipolar depression (n = 3462)	57.7 (50.1-66.0)	2.28 (2.00-2.59)	12.2 (8.9-16.3)	3.90 (2.91-5.11)
Anxiety disorder (n = 942)	38.4 (27.1-52.4)	1.67 (1.21-2.24)	12.1 (5.9-21.5)	3.65 (1.94-6.24)
Stress-related disorder (n = 1257)	75.8 (60.5-93.5)	2.57 (2.10-3.11)	10.1 (5.6-16.6)	3.46 (2.05-5.47)
Other (n = 2150)	60.0 (48.4-72.4)	2.92 (2.39-3.52)	10.9 (6.5-17.1)	4.47 (2.72-6.90)

∗ Using 5-year age-bands with England Standard Population as reference population.

† Using 5-year age-bands and comparing to local general population in Lambeth, Lewisham, Southwark, and Croydon (n = 161,603 people).

‡ Insufficient events.

### Incidence of Hospitalized Hip Fractures in the Year After Index Date

In total, 424 hip fractures occurred in the mental health cohort with an age-and-gender-standardized incidence rate of 13.7 (95% CI: 12.3-15.3) hip fractures per 1000 person-years. In the general population, 484 hip fractures occurred in 2011 with an age- and gender-standardized IRR of 2.78 (95% CI: 2.53-3.05) hip fractures per 1000 person-years. Comparing the patient and the general population the age- and gender-adjusted IRR for hip fractures was 4.18 (95% CI: 3.79-4.60). Examining the individual diagnostic groups (see Table 2), the highest age- and gender-standardized incidence rates for hip fractures were found in patients with a substance use disorder or dementia with rates above 15 hip fractures per 1000 person-years, and the lowest in patients with a psychotic disorder with a rate below 10 hip fractures per 1000 person-years. The highest increase of hip fractures compared with the general population was detected in people with a substance use disorder (IRR: 12.62), and the lowest in MCI (IRR: 2.45).

### Risk of First Hospitalized Fall by Diagnostic Group

In total, 15.2% (n = 3366) of the patient cohort had at least 1 hospitalized fall in the follow-up period, with a median interval until first fall or other censoring point of 2.1 years (interquartile range 0.7-44). Cox proportional hazard models assessing risk to first fall, comparing those with individual mental health conditions against the remainder of the patient sample, are presented in Table 3.

**Table 3:** Hazard Ratios (95% CIs) for Falls in Cox Regression Models for the Diagnostic Groups (Comparing Those With and Without the Respective Diagnosis)

Diagnostic Group	Adjustments in Model						
	Unadjusted	Age and Gender	All Demographic Factors	Demographics, Physical Illness, and Previous Fall	Demographics, Mental Health, and Functioning	Demographics and Medications	All Previous
Dementia (n = 7650)	**1.63 (1.53-1.75)**	**1.10 (1.02-1.18)**	**1.17 (1.09-1.26)**	**1.21 (1.12-1.30)**	**1.10 (1.01-1.21)**	**1.18 (1.10-1.28)**	**1.14 (1.04-1.25)**
Delirium (n = 2158)	**1.41 (1.25-1.59)**	**1.14 (1.01-1.28)**	1.11 (0.98-1.25)	1.03 (0.91-1.16)	1.04 (0.93-1.19)	1.10 (0.97-1.24)	0.99 (0.88-1.13)
MCI (n = 1026)	1.02 (0.86-1.20)	0.86 (0.73-1.01)	0.86 (0.73-1.02)	0.89 (0.75-1.05)	0.90 (0.76-1.07)	0.87 (0.74-1.03)	0.91 (0.77-1.08)
Substance use disorder (n = 1139)	0.92 (0.79-1.06)	**1.71 (1.46-2.01)**	**1.52 (1.29-1.78)**	**1.44 (1.23-1.69)**	**1.34 (1.13-1.59)**	**1.52 (1.29-1.78)**	**1.28 (1.08-1.52)**
Psychotic disorder (n = 1777)	**0.57 (0.50-0.66)**	**0.75 (0.65-0.86)**	**0.80 (0.69-0.93)**	**0.82 (0.71-0.95)**	**0.82 (0.71-0.96)**	**0.81 (0.69-0.95)**	0.87 (0.74-1.02)
Bipolar affective disorder (n = 542)	**0.74 (0.60-0.93)**	1.07 (0.85-1.33)	1.02 (0.82-1.28)	1.04 (0.83-1.30)	1.08 (0.86-1.36)	1.03 (0.82-1.29)	1.10 (0.88-1.38)
Unipolar depression (n = 3462)	**0.88 (0.80-0.97)**	0.97 (0.88-1.07)	0.94 (0.85-1.03)	0.93 (0.84-1.02)	1.01 (0.91-1.13)	0.90 (0.81-1.00)	0.97 (0.87-1.09)
Anxiety disorder (n = 942)	**0.68 (0.57-0.83)**	**0.76 (0.63-0.91)**	**0.71 (0.59-0.86)**	**0.74 (0.61-0.89)**	**0.78 (0.64-0.94)**	**0.70 (0.58-0.85)**	**0.77 (0.63-0.94)**
Stress-related disorder (n = 1257)	0.94 (0.80-1.11)	1.01 (0.86-1.19)	0.96 (0.81-1.13)	0.92 (0.78-1.08)	1.02 (0.86-1.21)	0.95 (0.81-1.12)	0.99 (0.83-1.17)

Boldface indicates statistical significance (*P* < .05).

### Risk of Hip Fracture by Diagnostic Group

Of the patient cohort, 5.2% (n = 1146) were hospitalized at least once for a hip fracture, with a median follow-up time to first hip fracture or other censoring point of 2.3 years (interquartile range 0.8-4.7). Cox proportional hazard models assessing risk to first hip fracture comparing those with individual mental health conditions against the remainder of the patient sample are presented in Table 4. After further adjustment for mental health, physical and functional difficulties, as well as prescribed medications, a significantly increased hazard remained for patients with dementia [hazard ratio (HR): 1.18], whereas patients with MCI had a lower risk (HR: 0.71).

**Table 4:** Hazard Ratios (95% CIs) for Hip Fractures in Cox Regression Models for the Diagnostic Groups (Comparing Those With and Without the Respective Diagnosis)

	Crude	Age and Gender	All Demographics	Demographics, Physical Illness, and Previous Fall	Demographics, Mental Health, and Functioning	Demographics and Medications	All Previous
Dementia (n = 7650)	**2.07 (1.84-2.32)**	**1.30 (1.15-1.47)**	**1.41 (1.24-1.59)**	**1.43 (1.26-1.63)**	1.15 **(**0.99-1.33) *P* = .07	**1.44 (1.27-1.64)**	**1.18 (1.01-1.37)**
Delirium (n = 2158)	**1.67 (1.37-2.03)**	**1.31 (1.07-1.59)**	**1.26 (1.04-1.54)**	1.22 (1.00-1.49)	1.10 **(**0.89-1.35)	1.20 (0.99-1.47)	1.07 (0.87-1.32)
MCI (n = 1026)	0.75 (0.54-1.04)	**0.62 (0.45-0.87)**	**0.63 (0.45-0.87)**	**0.64 (0.46-0.88)**	**0.69 (0.49-0.96)**	**0.64 (0.46-0.89)**	**0.71 (0.51-0.99)**
Substance use disorder (n = 1139)	**0.73 (0.55-0.97)**	**1.74 (1.30-2.34)**	**1.50 (1.11-2.01)**	**1.46 (1.09-1.97)**	**1.31 (0.95-1.78)**	**1.53 (1.14-2.07)**	1.32 (0.97-1.81)
Psychotic disorder (n = 1777)	**0.52 (0.40-0.67)**	**0.70 (0.54-0.91)**	0.83 (0.64-1.07)	0.84 (0.65-1.09)	0.89 (0.68-1.18)	**0.71 (0.54-0.93)**	0.83 (0.62-1.10)
Bipolar affective disorder (n = 542)	**0.55 (0.36-0.85)**	0.86 (0.56-1.33)	0.81 (0.52-1.25)	0.82 (0.53-1.27)	0.89 (0.57-1.37)	0.75 (0.48-1.16)	0.85 (0.54-1.31)
Unipolar depression (n = 3462)	**0.74 (0.63-0.88)**	**0.82 (0.69-0.97)**	**0.78 (0.65-0.92)**	**0.77 (0.65-0.91)**	0.97 (0.80-1.17)	**0.78 (0.65-0.94)**	0.95 (0.78-1.16)
Anxiety disorder (n = 942)	0.81 (0.60-1.10)	0.88 (0.65-1.20)	0.81 (0.60-1.09)	0.83 (0.61-1.12)	1.02 (0.75-1.39)	0.83 (0.61-1.13)	1.04 (0.76-1.42)
Stress-related disorder (n = 1257)	0.88 (0.66-1.18)	0.98 (0.73-1.30)	0.91 (0.68-1.21)	0.89 (0.66-1.19)	1.10 (0.82-1.48)	0.93 (0.70-1.24)	1.13 (0.84-1.52)

Boldface indicates statistical significance (*P* < .05).

## Discussion

To our knowledge, we describe the first representative clinical cohort study to assess the risk of hospitalized falls and hip fractures in older adults using mental health services. For the full cohort of more than 20,000 patients aged 60 years and older using a mental health service, we found incidence rates of 60.1 and 13.7 per 1000 person-years for falls for hip fractures, respectively, and a total 15.2% and 5.2% had a fall leading to hospitalization or hip fracture over median 2.1 and 2.3 years follow-up, respectively. When compared to data on more than 160,000 residents in the catchment population, age- and gender-standardized incidence was twice as high for hospitalized fall and 4 times as high for hip fractures. Although differences existed between the diagnostic subgroups, with incidence of falls more than 12 times as high in substance use disorder and almost 8 times as high in bipolar affective disorder, all 9 diagnostic groups of interest were associated with a higher incidence of fall and/or hip fractures than the general population. In further regression analyses within the patient sample, we found that those with dementia and substance use disorder diagnoses were at an increased risk of both hospitalized fall and hip fracture compared with the remainder of the sample. We found that even in the final model where we adjusted for medications associated with an increased risk of falls and fractures (eg, antidepressants and antipsychotics), the risk of falls and fractures was elevated in the aforementioned groups.

To date, the overwhelming majority of research in older adults has focussed on those with cognitive disorders; although this has indicated that people with dementia,^34^ delirium,^55^ and MCI^56^ are at increased risk of self-reported falls, there has been minimal use of representative health care data to investigate the most severe falls that lead to hospitalization in these cohorts. Similarly, although prior research has also suggested that older people with dementia,^14^ delirium,^57^ and MCI are at increased risk of hip fractures, sample sizes have been limited. Furthermore, our study is, to our knowledge, the first to establish that older people with dementia are at greatest risk of falls (HR 1.14) and hip fractures (HR 1.18) compared with those with other mental and cognitive disorders.

Beyond cognitive disorders, our findings suggest that older adults receiving mental health care for substance use disorders are also at particularly high risk of falls leading to hospitalization (IRR 6.72), followed by bipolar disorder (IRR 3.62), stress-related disorders (IRR 2.57), and clinical depression (IRR 2.28). A similar pattern was noted for substantially increased risk of hip fractures in each of these populations. Previous small-scale research relying on self-reported information on falls has suggested that older adults with substance use disorder and particularly alcohol use disorder are at increased risk.^58^^,^^59^ Potential reasons underlying these findings include intoxication or withdrawal states, as well as the influence of other key established risk factors for falls such as physical co/multimorbidity,^60^^,^^61^ inadequate nutrition, low physical activity and poor lower limb function,^62^ high smoking, and lower compliance with walking aids.^63^ Future research is clearly needed to identify and understand risk factors for older adults with substance use disorders for falls so that adequate falls prevention interventions can be developed.

To our knowledge, the current study provides the first representative data on falls leading to hospitalization and hip fractures in people with diagnosed bipolar disorder and stress-related disorders. Previous research has suggested that anticonvulsant use may be associated with increased risk of any fracture in older veterans with bipolar disorder.^64^ A previous systematic review of 3 studies suggested that bipolar disorder (at any age) was associated with an increased incidence of any fracture vs the general population.^65^ Our study advances the field demonstrating the increased risk of falls requiring hospitalization in older adults with bipolar disorder. For stress-related disorders, although previous research has suggested that some cases could arise as a consequence of both a fall and hip fracture and lead to worse outcomes,^66^ previous research has also reported that older adults with depressive symptoms, that is, a potentially related group, have an increased risk of hip osteoporosis,^30^ self-report falls,^38^ and fractures.^67^ Our data indicate that people with clinical depression specifically have a 2.2 and 3.9 increased risk of hospitalized falls and hip fractures, respectively.

The underlying reasons for the increased risk of falls and hip fractures are likely complex, but include increased physical comorbidity, side effects of common psychotropic medication, vulnerability from lifestyle risk factors (eg, increased smoking, low physical activity, and inadequate diet), potentially diagnostic overshadowing, and difficulty accessing mainstream falls prevention and bone health care pathways.^68^ To compound this, current evidence-based guidelines and randomized controlled trials for the prevention of falls and hip fractures typically exclude people with mental and cognitive health conditions.^69^^,^^70^ Although there have recently been some concerted efforts to reduce falls and hip fractures and improve their rehabilitation in people with dementia and MCI,^71^ those with mental health and substance use disorders remain at risk of being overlooked. Given that older adults with substance use disorders and mental disorders appear at greatly increased risk of both falls and hip fractures, concerted efforts are required to understand and prevent these outcomes and develop better integrated models of care.

Although the findings are novel, some important limitations should be noted. First, it was not possible to collect information on all potential confounding factors (eg, pre-fall/hip fracture mobility, balance), which are key risk factors for falls.^3^ Second, the study relied only on falls and fractures leading to a hospital admission, which is the most severe end of the spectrum for these outcomes. It is likely that the figures for falls are substantial underestimates of the true risk of falls in older adults with mental disorders. Third, some of the mental disorder groups had relatively small numbers of people, although they were substantially higher than in previous literature.

## Conclusions and Implications

Our novel data suggest that older adults using mental health services are at substantially increased risk of falls leading to hospitalization and hip fractures compared with the general population. There is a particularly high risk of falls and hip fractures in those with substance use disorders, bipolar disorder, dementia, MCI, and delirium. Future interventions and care pathways are needed to identify older adults with mental health and cognitive disorders at risk of falls and hip fractures and to prevent these adverse outcomes.
